# Remifentanil requirement for i-gel insertion is reduced in male patients with Parkinson’s disease undergoing deep brain stimulator implantation: *an up-and-down sequential allocation trial*

**DOI:** 10.1186/s12871-022-01735-0

**Published:** 2022-06-24

**Authors:** Wenjun Meng, Fang Kang, Meirong Dong, Song Wang, Mingming Han, Xiang Huang, Sheng Wang, Juan Li, Chengwei Yang

**Affiliations:** Department of Anesthesiology, The First Affiliated Hospital of USTC, Division of Life Sciences and Medicine, University of Science and Technology of China, Hefei, 230036 China

**Keywords:** Parkinson’s disease, Pharmacology, Remifentanil, I-gel insertion, Deep brain stimulator implantation

## Abstract

**Background:**

Laryngeal mask airways have been widely used in clinical practice. The aim of this study was to investigate whether the remifentanil requirement for facilitation of i-gel insertion in Parkinson’s disease (PD) patients undergoing deep brain stimulation (DBS) surgery was different from that in non-PD (NPD) patients undergoing intracranial surgery.

**Study design:**

An up-and-down sequential allocation trial.

**Methods:**

Male patients aged between 40 and 64 years old were enrolled. The first patient in each group (PD and NPD) group received an effect-site concentration (Ce) of remifentanil (Minto pharmacokinetic model) of 4.0 ng.ml^−1^ during a target-controlled infusion (TCI) of 3.5 μg.ml^−1^ propofol (Marsh pharmacokinetic model). The next dose of remifentanil was determined by the response of the previous patient. The Ce of remifentanil required for i-gel insertion in 50% of patients (EC_50_) was estimated by the modified Dixon’s up-and-down method and by probit analysis.

**Results:**

The PD group included 24 patients and the NPD group included 23. The EC_50_ of remifentanil for i-gel insertion during a TCI of 3.5 μg.ml^−1^ propofol estimated by the modified Dixon’s up-and-down method in PD patients (2.38 ± 0.65 ng.ml^−1^) was significantly lower than in NPD patients (3.21 ± 0.49 ng.ml^−1^) (*P* = 0.03). From the probit analysis, the EC_50_ and EC_95_ (effective Ce in 95% of patients) of remifentanil were 1.95 (95% CI 1.52–2.36) ng.ml^−1^ and 3.12 (95% CI 2.53–5.84) ng.ml^−1^ in PD patients and 2.85 (95% CI 2.26–3.41) ng.ml^−1^ and 4.57 (95% CI 3.72–8.54) ng.ml^−1^ in NPD patients, respectively.

**Conclusions:**

The remifentanil requirement for successful i-gel insertion is reduced in male PD patients undergoing DBS implantation during propofol TCI induction. Clinicians should closely monitor the remifentanil requirement in patients with PD.

**Trial registration:**

Registered at http://www.chictr.org.cn (ChiCTR1900021760).

## Background

Of all the neurological disorders included in the *Global Burden of Diseases, Injuries, and Risk Factors* (GBD) 2015 [1], Parkinson's disease (PD) was the fastest growing. In 2016, 6.1 million individuals worldwide suffered from PD compared with 2.5 million in 1990 [2]. This increase was due to increasing numbers of older people and environmental factors. The incidence of PD increases rapidly with age after the age of 50 years, and its prevalence peaks at between 85 and 89 years of age [2]. The symptoms of PD include rest tremor, muscular rigidity, bradykinesia, and loss of postural reflexes, which seriously affect the quality of life of PD patients [3]. Concern has been raised about anaesthetic management in PD patients because of the possibility of interaction between anaesthetics and anti-PD medications or Parkinsonian symptoms [4, 5]. However, there are few studies focusing on the pharmacodynamic changes of anaesthetics in PD patients [6, 7].

Deep brain stimulation (DBS) is approved for people who have had PD for at least four years and who benefit from medication but have motor complications, such as significant ‘off’ time and/or dyskinesia (uncontrolled, involuntary movements). Which type of anaesthesia should be used to perform stereotactic procedures for insertion of DBS electrodes currently remains controversial [8, 9]. In our hospital, surgeons usually perform insertion of DBS electrodes under local anaesthesia. After confirmation of proper positioning of the DBS electrodes by magnetic resonance imaging examination, we conduct general anaesthesia to place the pulse generator in the upper chest.

A previous study demonstrated that remifentanil concentrations required for inhibiting responses to tracheal intubation and skin incision are reduced in PD patients compared with non-PD (NPD) patients due to the degenerative brain [6]. Laryngeal mask airways have been widely used in clinical practice, as they are effective and safe for ventilating patients and less invasive than endotracheal tubes [10, 11]. However, the effect-site concentration (Ce) of remifentanil required for the successful insertion of i-gel combined with propofol in patients with PD is unknown. Furthermore, the issue of whether PD patients require a lower dose of remifentanil for i-gel insertion than do NPD patients is of interest. Thus, the primary purpose of this study was to compare the effective Ce in 50% of patients (EC_50_) of remifentanil for facilitation of i-gel insertion between PD and NPD patients.

## Methods

Our study was an up-and-down sequential allocation trial. This trial was approved by the Clinical Research Ethics Committee of The First Affiliated Hospital of University of Science and Technology of China, Anhui, China (2018KY40, Chairperson Prof. Zuojun Shen) on 8 December 2018 and registered at http://www.chictr.org.cn (ChiCTR1900021760). Trial registration: Chinese Clinical Trial Registry, ChiCTR1900021760. Registered 23 April 2019, http://www.chictr.org.cn/edit.aspx?pid=36620&htm=4. This study was performed from April 2019 to December 2019. Written informed consent was obtained from all subjects. The inclusion criteria were as follows: (1) male, (2) 18–64 years old, (3) body mass index (BMI) 18.5–24.9 kg/m^2^, and (4) American Society of Anesthesiologists (ASA) physical status I–III. The exclusion criteria were as follows: (1) predicted difficult airway, reactive airway disease, (2) alcohol or drug abuse, (3) a risk of gastric aspiration, and (4) patients with diseases that affected their neurological function, such as epilepsy and brain tumours in the eloquent brain area. The PD group consisted of a group of patients with PD undergoing bilateral DBS insertion and pulse generator placement. In contrast, the NPD group consisted of another group of patients without PD who were scheduled for intracranial surgery, such as patients with hemifacial spasm or small (less than 30 mm in diameter) supratentorial brain tumours. PD patients stopped taking anti-PD drugs the night before surgery. As mentioned above, after performing DBS electrode insertion in the proper site under local anaesthesia, we conducted general anaesthesia to place the pulse generator in PD patients.

All patients received no premedication and were routinely fasted before surgery. A 20G venous cannula was sited. Then, lactated Ringer’s solution (10 ml.kg^−1^) was infused at a rate of 10 ml.kg^−1^.h^−1^. Upon arrival in the operating room, patients were preoxygenated with 100% oxygen for three minutes. Routine monitoring was used, including electrocardiogram, pulse oxygen saturation, noninvasive arterial pressure and end-tidal CO_2_ concentration (EtCO_2_). The depth of anaesthesia was monitored using a bispectral index (BIS) monitor (BIS VISTA™ monitor, Aspect Medical Systems, Norwood, MA). Anaesthesia was induced with propofol and remifentanil by using a two-channel target-controlled infusion (TCI) pump (CP-730TCI; Inc., Beijing SLGO, China). The effect-site concentration (Ce) of propofol was set at 3.5 μg.ml^−1^ under the Marsh pharmacokinetic model. Five minutes after the administration of propofol, remifentanil was intravenously infused at the predetermined Ce under the Minto pharmacokinetic model. Assisted respiration was conducted manually to maintain the EtCO_2_ concentration within the range of 35–45 mmHg. We did not use a muscle relaxant during the i-gel insertion. Five minutes after administration of remifentanil, an experienced anaesthesiologist performed the i-gel (Intersurgical Incorporated, NY and UK) insertion according to the manufacturer’s recommendations (size 3 for 30 kg ≤ body weight ≤ 60 kg, size 4 for 60 kg < body weight ≤ 90 kg, and size 5 for body weight > 90 kg). The anaesthesiologist who performed or evaluated the conditions of i-gel insertion was blind to the remifentanil concentration used.

The Ce of remifentanil required for i-gel insertion in 50% of patients (EC_50_) was determined by using a modified Dixon’s up-and-down method based on altering the test space [12, 13]. Compared with the original Dixon’s up-and-down method, this method increases the precision of the final estimator and reduces the mean squared error under normal tolerance distribution [13, 14]. In the present study, the concentrations of propofol and remifentanil and the step size of the dose of remifentanil were chosen according to the results of previous work by Kim et al. [12]. The first patient received a remifentanil Ce of 4.0 ng.ml^−1^. If the i-gel insertion was a success, then the target Ce for the next patient was decreased by a step of 1 ng.ml^−1^. In contrast, if insertion was a failure, the target Ce for the next patient was increased by 1 ng.ml^−1^. The first stage consisted of an up-and-down sequence of steps of 1 ng.ml^−1^ until three ‘negative–positive’ (i.e. failure-to-success of i-gel insertion) crossovers were obtained. Then, the step size of remifentanil concentration was reduced to 0.5 ng.ml^−1^. The study was continued until six ‘negative–positive’ crossover pairs occurred.

Failure was defined as patient movement, including coughing, bucking, straining, laryngospasm or gross purposeful movement, at the time of i-gel insertion or within one minute of insertion. Also, significant resistance to mouth opening (Muzi score higher than 2, as previously described [15, 16]), was classified as a failed insertion. If a failed insertion occurred, we intravenously injected additional doses of propofol or remifentanil and then reattempted i-gel insertion. One minute after the successful i-gel insertion, 0.6 mg.kg^−1^ rocuronium was intravenously administered. An endotracheal tube was inserted through the conduit under the guidance of the 2.8 mm Flexible Intubation Videoscope (TIC-SD-II; UESCOPE, China) (using ID 6.0 mm for a size 3 i-gel, or ID 6.5 mm for a size 4 i-gel) in NPD patients based on our previous study [17].

We recorded the patient’s vital signs data including heart rate (HR), mean arterial pressure (MAP), and BIS at baseline (just before anaesthesia induction), at one, three and five minutes after administration of propofol and remifentanil and one minute after i-gel insertion. Ephedrine (6–10 mg) was administered intravenously when MAP was less than 60 mmHg or decreased by more than 30% of the baseline value. Atropine (0.5 mg) was intravenously injected when HR was less than 45 beats.min^−1^ or decreased by more than 30% of the baseline value. All patients were interviewed in the postoperative anaesthetic care unit to assess whether intraoperative awareness occurred.

The calculation of sample size is based on Kim’s research and our pilot study [12]. We hypothesized that the EC_50_ of remifentanil for i-gel insertion in PD and NPD patients were 2.4 ng.ml^−1^ and 3.2 ng.ml^−1^, respectively. The standard deviation was 0.8. Thus, 17 patients per group were required to achieve a power of 80% with a type 1 error of 0.05. Allowing for a 15% drop-out rate, at least 20 patients were enrolled in each group.

The statistical analysis was performed using SPSS version 19.0 software (SPSS Inc., Chicago, IL). For continuous variables, data are expressed as the mean ± standard deviation. For categorical variables, data are expressed as the number (percentage) of patients. After obtaining six ‘negative–positive’ crossover points, the average of the midpoint concentration for each independent pair of patients was calculated to determine the EC_50_ of remifentanil required for i-gel insertion in each group. A probit analysis was also used to process the data of the Dixon’s up-and-down method. Then we derived the EC_50_ and the effective Ce in 95% of patients (EC_95_) with a 95% confidence interval (CI). A Student *t* test or chi-square analysis was used for comparison between the two groups. The hemodynamic changes and BIS values were compared using repeated measures analysis of variance. A *P* value < 0.05 was defined as statistically significant.

## Results

**Fig. 1:**
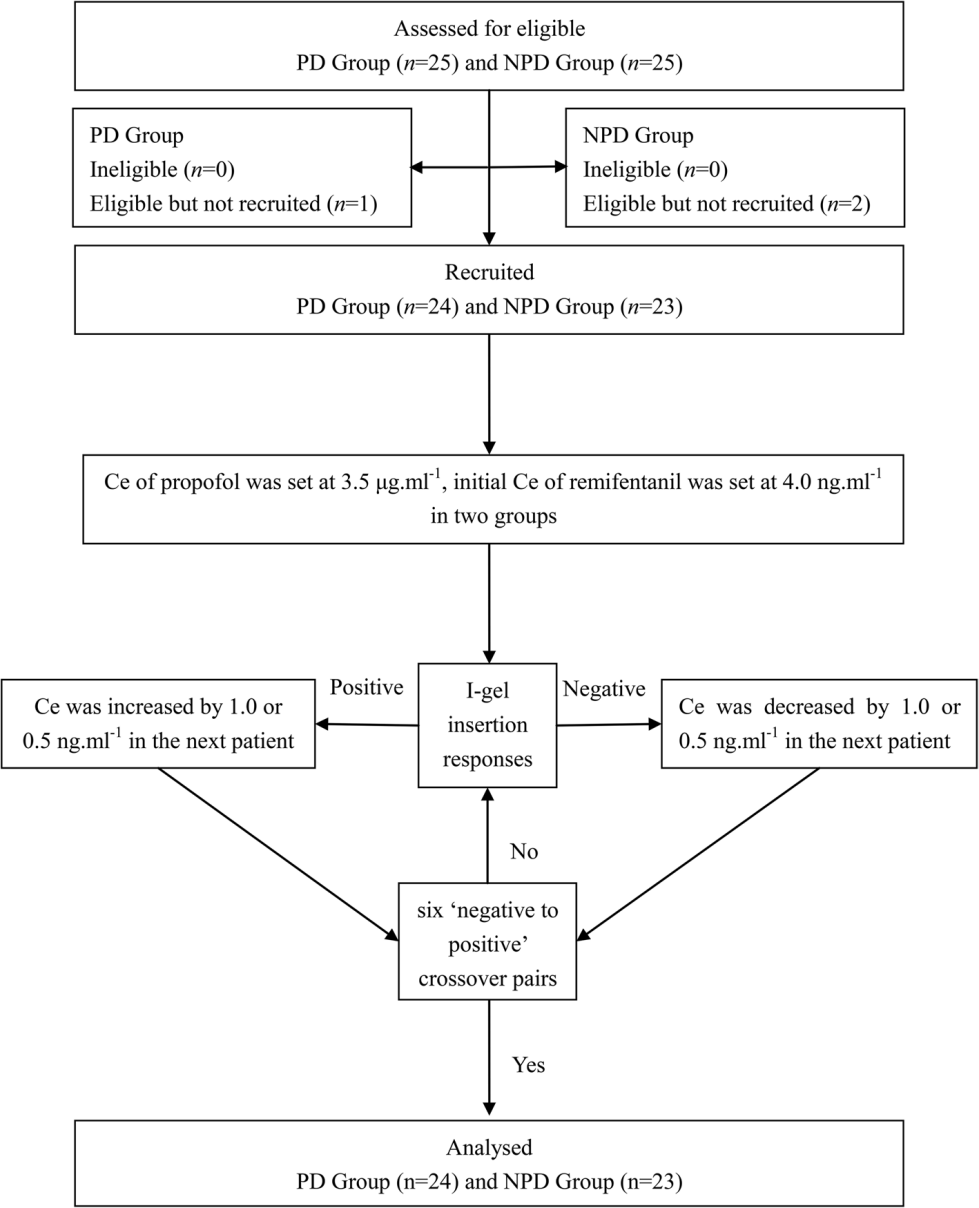
Flow diagram for the Dixon’s up-and-down method. PD, Parkinson’s disease; NPD, non-Parkinson’s disease; Ce, effect-site concentration

A total of 47 patients were enrolled in this study, with 24 in the PD group and 23 in the NPD group (Fig. 1). The mean duration of PD was 8.1 ± 2.7 years. According to the work by Tomlinson and colleagues [18], the dosage of each anti-Parkinson drug was transformed to a levodopa equivalent dose. Thus, the mean dosage of the levodopa was 615 ± 276 mg/d in PD patients. Patients’ general demographic parameters are shown in Table 1. The age, height, weight, BMI and ASA physical status were comparable between the PD and NPD groups.

**Table 1:** Demographic data of patients included

Parameters	PD Group (*n* = 24)	NPD Group (*n* = 23)	*P*
Age (y)	56.4 ± 5.8	56.3 ± 5.0	0.943
Height (cm)	168.7 ± 5.1	169.4 ± 3.8	0.608
Weight (kg)	66.3 ± 9.8	70.1 ± 8.3	0.110
BMI (kg.m^−2^)	23.2 ± 2.7	24.6 ± 2.7	0.088
ASA physical status (II/III)	19/5	17/6	0.671

Data are presented as mean ± SD or number

*PD* Parkinson’s disease, *NPD* non- Parkinson’s disease, *BMI* Body mass index, *ASA* American Society of Anesthesiologists

**Fig. 2:**
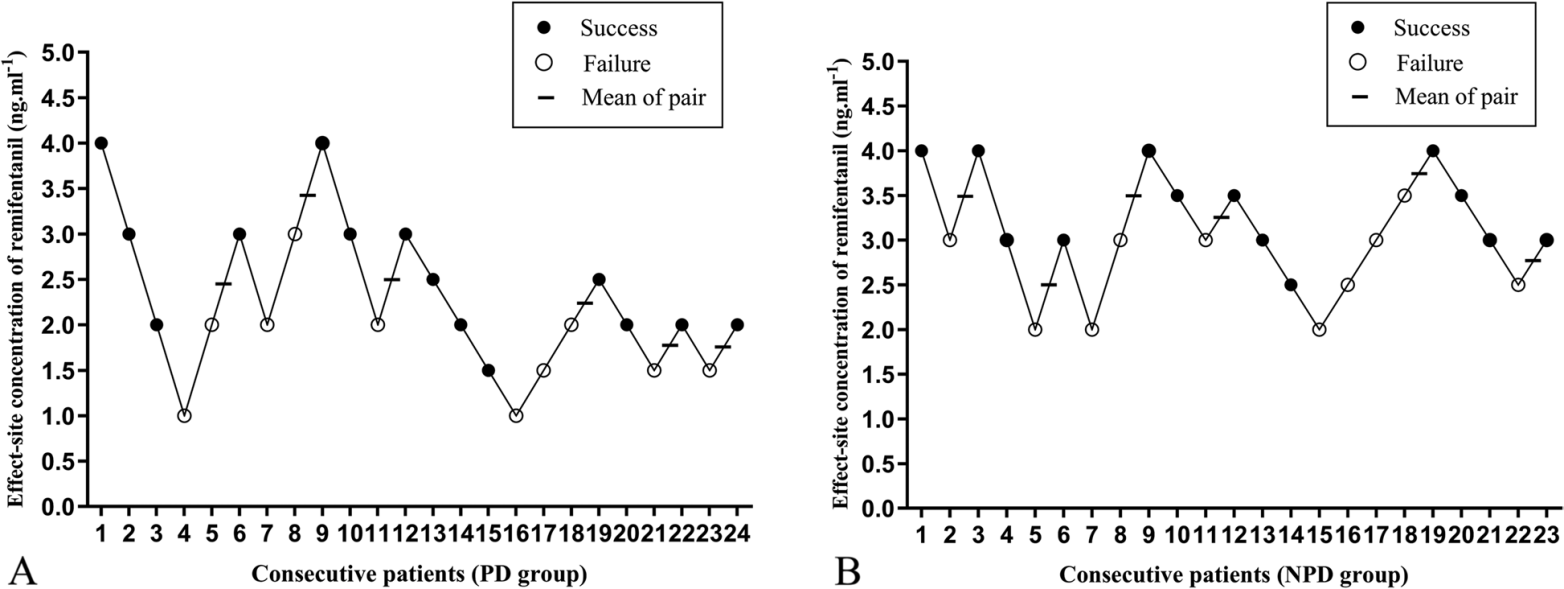
Patient responses to i-gel insertion. A successful insertion dose is denoted by a solid circle; a failed insertion dose is denoted by an open circle; horizontal bars represent crossover midpoints (failure-to-success). PD, Parkinson’s disease; NPD, non-Parkinson’s disease

**Fig. 3:**
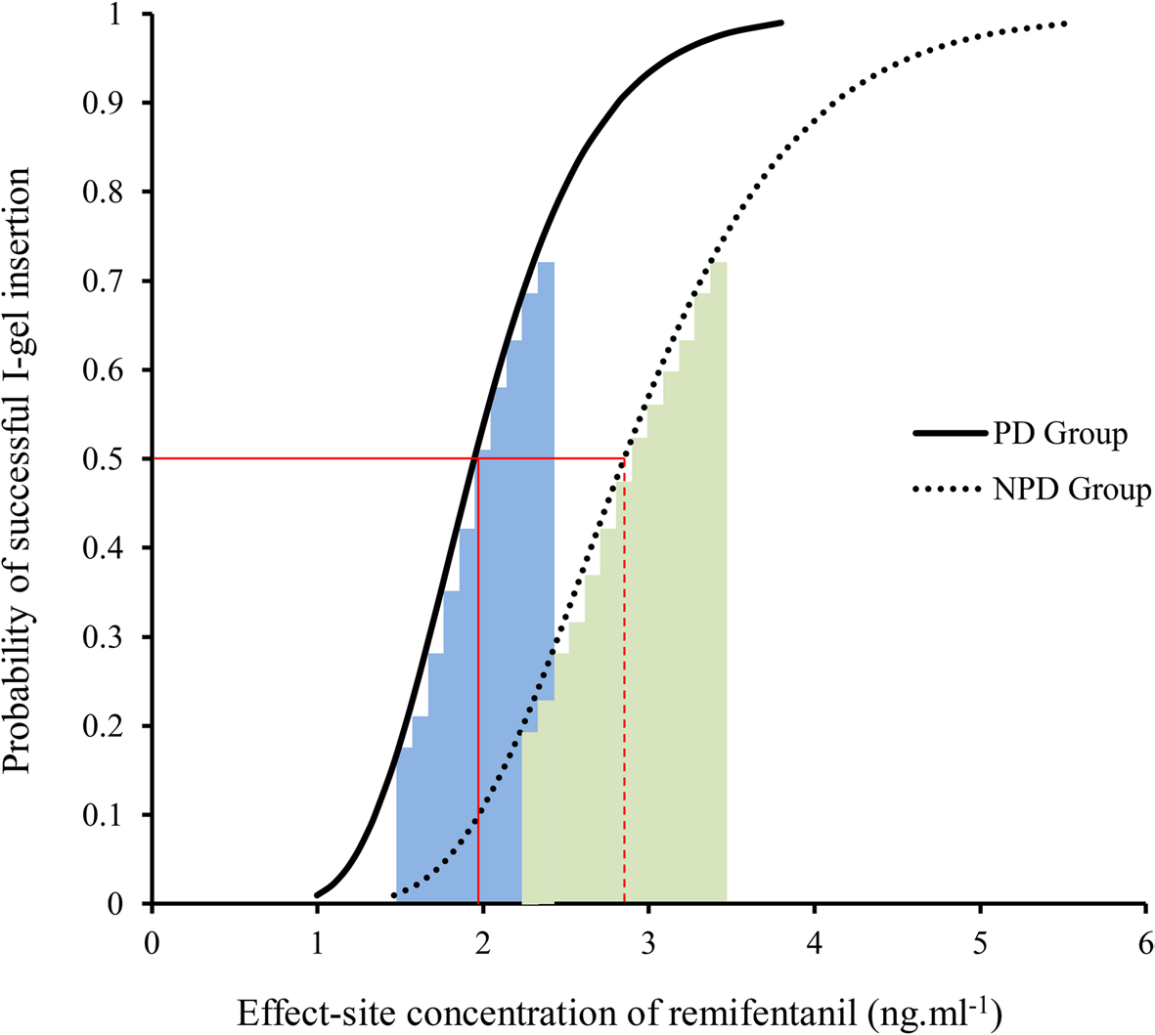
Dose–response curve from the probit analysis. PD, Parkinson’s disease; NPD, non-Parkinson’s disease. The shaded areas indicate the confidence intervals of the EC_50_ (blue for PD group, green for NPD group)

The sequences for success and failure of i-gel insertion in the two groups are shown in Fig. 2. The EC_50_ of remifentanil required for i-gel insertion during a TCI of 3.5 μg.ml^−1^ propofol estimated by the modified Dixon’s up-and-down method in PD patients (2.38 ± 0.65 ng.ml^−1^) was significantly lower than in NPD patients (3.21 ± 0.49 ng.ml^−1^) (*P* = 0.03). From the probit analysis, EC_50_ and EC_95_ of remifentanil were 1.95 (95% CI 1.52–2.36) ng.ml^−1^ and 3.12 (95% CI 2.53–5.84) ng.ml^−1^ in PD patients and 2.85 (95% CI 2.26–3.41) ng.ml^−1^ and 4.57 (95% CI 3.72–8.54) ng.ml^−1^ in NPD patients, respectively (Fig. 3 and Table 2).

**Table 2:** Remifentanil requirement for I-gel insertion during a TCI of propofol in PD group and NPD group

	**PD Group (*n* = 24)**	**NPD Group (*n* = 23)**
Dixon’s method EC_50_ (ng.ml^−1^)	2.38 ± 0.65	3.21 ± 0.49
Probit analysis		
EC_50_ (ng.ml^−1^)	1.95 (1.52–2.36)	2.85 (2.26–3.41)
EC_95_ (ng.ml^−1^)	3.12 (2.53–5.84)	4.57 (3.72–8.54)

Data from Dixon’s up-and-down method are presented as mean ± SD. Date from probit analysis are EC_50_ or EC_95_ with (95% confidence intervals)

*EC*_*50*_ effective effect-site concentration in 50% of patients, *EC*_*95*_ effective effect-site concentration in 95% of patients

**Fig. 4:**
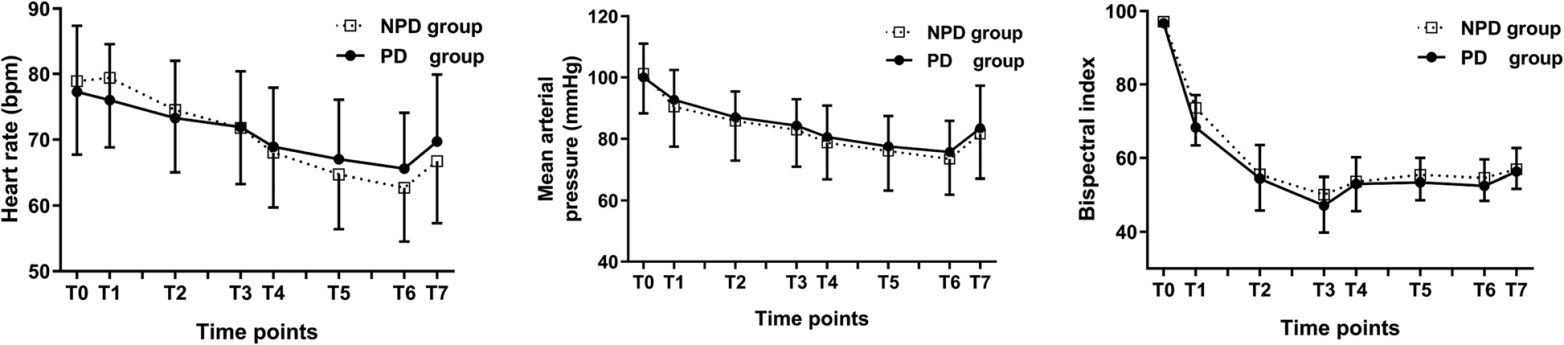
Changes in HR, MAP and BIS during i-gel insertion. Data are presented as mean ± SD. PD, Parkinson’s disease; NPD, non- Parkinson’s disease. T0, baseline; T1, 1 min. after propofol infusion; T2, 3 min. after propofol infusion; T3, 5 min. after propofol infusion and starting time of remifentanil infusion; T4, 1 min. after remifentanil infusion; T5, 3 min. after remifentanil infusion; T6, 5 min. after remifentanil infusion; T7, 1 min. after i-gel insertion

Changes in HR, MAP and BIS during the i-gel insertion are shown in Fig. 4. Compared with baseline values, HR three minutes after propofol infusion, MAP after propofol infusion and BIS after propofol infusion were significantly decreased in both groups (*P* < 0.05). However, there was no difference in HR, MAP and BIS at each time point between the two groups. No patients required treatment for significant hypotension or bradycardia. Intraoperative awareness did not occur in this study.

## Discussion

Using a modified Dixon’s up-and-down method, this study demonstrated that the EC_50_ of remifentanil required for i-gel insertion in male patients was significantly lower in the PD group (2.38 ± 0.65 ng.ml^−1^) than in the NPD group (3.21 ± 0.49 ng.ml^−1^) during propofol anaesthesia without neuromuscular blockade.

The Dixon’s up-and-down method is widely used to determine the median effective concentration of a drug [19]. In the current study, we used a modified Dixon’s up-and-down method based on altering the test space. Jung et al. demonstrated that this method tends to be somewhat better than the original up-and-down method in terms of the mean squared error under normal tolerance distribution [14]. Patients’ general demographic parameters including age, height, weight and ASA physical status were comparable between the two groups, making our results more convincing. In our centre, of the 124 individuals undergoing DBS surgery in 2018, 90 were younger than 65 years. Moreover, the age factor may influence the remifentanil requirement [20, 21]. Therefore, middle-aged patients with PD were enrolled in our study. The average age of PD patients in our study (56.4 ± 5.8 years) was similar to the average age of patients in previous work by Wang et al. (57.4 ± 9.1 years) in China [6], which may indicate that the age of PD patients tends to be young. In addition, previous studies found that the EC_50_ of remifentanil for laryngeal mask airway (LMA) insertion is lower during propofol in women (2.18 ± 0.35 ng.ml^−1^) than in men (2.82 ± 0.53 ng.ml^−1^). There are differences between male and female patients in the following respects: sensitivity to opioid receptor agonists [22], suppression of the cough reflex or airway reactivity by opioids [23], and the activity of nonspecific esterase [24]. These may be possible reasons for the sex difference in the use of remifentanil for i-gel insertion [25]. Therefore, we only enrolled male patients in this study to reduce the interference of sex factors.

The present study demonstrated that the EC_50_ of remifentanil for i-gel insertion during a TCI of 3.5 μg.ml^−1^ propofol in NPD patients was 3.21 ± 0.49 ng.ml^−1^, slightly higher than that calculated by Kim et al. (3.04 ± 0.49 ng.ml^−1^) [12]. This difference may be because they enrolled patients of both sexes. Joe et al. [25] reported that the EC_50_ of remifentanil for LMA insertion in men was 2.82 ± 0.53 ng.ml^−1^ with propofol TCI at 3.5 μg.ml^−1^. In another Asian population, the EC_50_ of remifentanil for i-gel insertion in female patients was 1.58 ± 0.41 ng.ml^−1^ with a propofol Ce of 5 μg.ml^−1^ [26]. These differences may be caused by differences in study design, such as the use of lidocaine, differing sex of patients and different concentrations of propofol.

There is a difference between the ED_50_ from the modified Dixon’s up-and-down method and that from the probit analysis. As we know, calculating the average of the midpoint dose for each crossover pair in up-and-down studies is a widely used method for determining ED_50_ [25–28]. A previous study found that the parameter estimate of probit analysis is biased and the CIs of the ED_50_ may be unrealistically narrow [29]. This seemed to be a consequence of the nonindependence of assigned dose values. This may cause the difference between the results of the two methods.

General anaesthesia is more and more often required for PD patients as the DBS surgery is very burdensome. Patients frequently experience pain during stereotactic frame placement and during the surgery despite the use of local anaesthesia. Furthermore, patients must endure a prolonged period of off-medication symptoms while experiencing anxiety and exhaustion due to clinical testing [30, 31]. Unfortunately, most studies have focused on the influence of anaesthetics on PD symptoms or anti-PD medications. Only very few studies have been performed to investigate the abnormal pharmacodynamic characteristics of anaesthetics in PD patients due to the potential intracranial lesions [6, 7]. An inappropriate use of anaesthetic drugs may affect the practice of enhanced recovery after surgery in these patients.

Our results showed that the EC_50_ of remifentanil for i-gel insertion was reduced in PD patients. The explanation for these results may be found in two factors: suppression of the cough reflex or airway reactivity and a reduced requirement for analgesics. Suppression of the cough reflex or airway reactivity is one of the most important factors for successful i-gel insertion without neuromuscular blockade. Fontana and colleagues [32] found a higher cough threshold for patients with PD. Troche et al. [33] observed that various components of the cough behaviour including a consistent two-cough sequence, total number of coughs and urge-to-cough ratings in response to 200 μm capsaicin were impaired in PD patients, particularly in those with dysphagia. Accordingly, cough reflex impairment may facilitate i-gel insertion in PD patients. Several studies found that PD patients had increased pain sensitivity compared to healthy controls in response to noxious experimental stimulation, while other studies failed to find this effect. Pain is one of the most frequent nonmotor impairments in PD and is hypothesized to be associated with altered nociceptive pain processing [34, 35]. These findings may indicate that PD patients need more analgesics. However, systematic reviews also demonstrated that abnormal pain thresholds in PD patients were significantly diminished when tested on dopaminergic medications compared to when they were not on medications [36, 37]. As all the PD patients undergoing DBS surgery enrolled in our study had been taking dopaminergic medications for a long time, they tended to have relatively normal pain sensitivity. In addition, dopamine denervation or levodopa-induced increase in opioid transmission in PD might be another reason for the present results. The enhanced opioid transmission includes preproenkephalin and enkephalin precursor, both of which are believed to contribute to the mechanism of analgesia [38]. It remains to be clarified whether PD patients have a reduced requirement for analgesics.

There are several limitations in our study. First, the concentrations of propofol and remifentanil used were calculated by using pharmacokinetic models, not actual plasma concentrations obtained from sampling patients’ blood. However, the Marsh pharmacokinetic model for propofol and the Minto model for remifentanil have been commonly used with acceptable levels of bias and accuracy in the clinical setting [39–41]. Second, a previous study demonstrated that the propofol requirement for induction of unconsciousness is reduced in PD patients compared with NPD patients. However, BIS values at each time point were comparable between the two groups, indicating that patients in both groups were under the same depth of anaesthesia. Third, the sample size in our study was relatively small. But a cohort size of 20 to 40 patients is generally acceptable based on up-and-down methodology [19]. Fourth, we enrolled only middle-aged male patients in our study. More research may be needed before recommending changes to clinical practice.

In conclusion, the EC_50_ of remifentanil for successful i-gel insertion is reduced in male patients with PD undergoing DBS and pulse generator placement during propofol anaesthesia. Clinicians should closely monitor the remifentanil requirement in patients with PD.

## Data Availability

The datasets used and analysed during the current study are available from the corresponding author on reasonable request.

## Abbreviations

PD: Parkinson’s disease
DBS: Deep brain stimulation
Ce: Effect-site concentration
ASA: American Society of Anesthesiologists
EtCO_2_: End-tidal CO_2_ concentration
BIS: Bispectral index
TCI: Target-controlled infusion
HR: Heart rate
MAP: Mean arterial pressure
EC_50_: Effective effect-site concentration in 50% of patients
EC_95_: Effective effect-site concentration in 95% of patients
